# Zika virus infection in 18 travellers returning from Surinam and the Dominican Republic, The Netherlands, November 2015–March 2016

**DOI:** 10.1007/s15010-016-0906-y

**Published:** 2016-05-21

**Authors:** Janneke W. Duijster, Abraham Goorhuis, Perry J. J. van Genderen, Leo G. Visser, Marion P. Koopmans, Johan H. Reimerink, Martin P. Grobusch, Annemiek A. van der Eijk, Johannes H. C. T. van den Kerkhof, Chantal B. Reusken, Susan J. M. Hahné, The Dutch ZIKV study team

**Affiliations:** 1 Centre for Infectious Disease Control, National Institute for Public Health and the Environment (RIVM) Bilthoven, Antonie van Leeuwenhoeklaan 9, 3721 MA Bilthoven, The Netherlands; 2 Division of Internal Medicine, Department of Infectious Diseases, Center of Tropical Medicine and Travel Medicine, Academic Medical Center, University of Amsterdam, Meibergdreef 9, A01-330, 1105 AZ Amsterdam, The Netherlands; 3 Institute for Tropical Diseases, Harbour Hospital, Rotterdam, Haringvliet 72, 3011 TG Rotterdam, The Netherlands; 4 Department of Infectious Diseases, Leiden University Medical Centre, Leiden, Albinusdreef 2, 2333 ZA Leiden, The Netherlands; 5 Department of Viroscience, WHO Collaborating Centre for Arbovirus and Haemorrhagic Fever Reference and Research, Erasmus MC, Rotterdam, Wytemaweg 80, Ee 1726, 3015 CN Rotterdam, The Netherlands

**Keywords:** Imported viral diseases, Netherlands, Zika virus, Travel, Surinam, Dominican Republic

## Abstract

**Purpose:**

We report 18 cases of confirmed Zika virus (ZIKV) infection in travellers returning to the Netherlands from Surinam (South America, bordering northern Brazil) and the Dominican Republic.

**Methods:**

In a multi-centre study, we collected epidemiological, virological and clinical characteristics, as well as data on travel history, underlying illness and laboratory results of the 18 imported ZIKV infection cases using a standardised form.

**Results:**

Most cases had a self-limiting course of disease, two patients developed complications, one had Guillain–Barré and another had severe thrombocytopenia. Four patients had underlying illness. One of the reported cases was pregnant. Three of 13 patients tested had a weak-positive result for dengue IgM. The majority of patients were born in Suriname and/or visiting friends and relatives (VFR).

**Conclusions:**

Providing pre-travel advice among travellers, especially VFR travellers, is needed to enhance the use of preventive measures against ZIKV infection. Further evidence on health risks associated with ZIKV infection is urgently needed.

## Introduction

Zika virus (ZIKV) is a flavivirus transmitted by *Aedes* mosquitoes which has recently emerged in South and Central America, causing large numbers of human infections. After discovery of the virus in 1947 in Uganda, serological evidence of virus circulation has been reported from various African and Asian countries. However, up to 2007, ZIKV was not associated with epidemics. To date, two lineages of ZIKV are known, an African and an Asian lineage. The latter caused the first outbreak of ZIKV on Yap Island and adjoining islands in the Federate State of Micronesia in 2007 [1]. Subsequently, outbreaks occurred in French Polynesia, New Caledonia, the Cook Islands and Easter Island, before ZIKV was first noticed in Brazil in May 2015 [2]. Currently, ZIKV infections have been reported from a large number of South American countries and territories, including several Caribbean islands [3]. In Surinam, a country in South America bordering northern Brazil, the first case was confirmed in November 2015 [2]. In the three Dutch Caribbean municipalities, the first case of endemic ZIKV infection was reported from Bonaire, on February 15. As of March 8, no endemic cases have been reported from the other two Dutch Caribbean municipalities (Sint Eustatius and Saba). However, the three Dutch overseas territories (Curacao, St. Maarten and Aruba) have reported multiple autochthonous cases [3].

About 80 % of ZIKV infections remain asymptomatic; symptoms are generally mild and include a maculopapular rash, fever, headache and conjunctivitis [2]. Since late 2015, there are increasing concerns about the probable association between ZIKV infection and the development of congenital malformations. In Brazil, the reported prevalence of newborns with microcephaly increased sharply since the start of the outbreak in May 2015 [4]. Several case reports of congenital malformations where ZIKV has been detected have been published [4, 5]. An additional concern is the increase in Guillain–Barré syndrome (GBS) which has been reported in multiple affected countries including Brazil and Ecuador among people recently infected with ZIKV [4]. Recently, a case–control study in Polynesia found a strong association between ZIKV infection and GBS, suggestive of a causal relation [6]. Apart from the main mode of transmission by mosquitoes, there is evidence of other routes of transmission such as sexual transmission through blood transfusion [4]. The possibility of transmission via urine and saliva remains unclear.

On the 1st of February 2016, the WHO declared the recent clusters of microcephaly and neurological disorders and their possible association with ZIKV (in the Americas) to be a Public Health Emergency of International Concern (PHEIC). In this declaration, countries are requested to share clinical, virological and epidemiological data regarding microcephaly incidence, neurological disorders and ZIKV transmission with the WHO, to facilitate sharing of knowledge, guide future actions for control efforts and research on an international level [7]. Along with the spread of the virus in the Americas, there is an increasing risk of introduction of ZIKV into Europe as there is intense travel activity between Europe and the affected countries. Introduction of ZIKV into Europe by travellers from the Americas has been described in Italy and France in 2015 and 2016. These were all cases of self-limiting illness [8, 9]. In this rapid communication, we describe epidemiological, virological and clinical characteristics of 18 reported ZIKV infection cases in the Netherlands. Seven of the earliest cases have been briefly described elsewhere before a standardised comprehensive nation-wide registry became functional [10–12].

## Methods

ZIKV infection is currently not mandatory notifiable in the Netherlands. Cases are defined by a ZIKV-positive quantitative real-time polymerase chain reaction (qRT-PCR) test result in an individual with symptoms. Additional data on travel history, underlying illnesses, use of precautions to avoid mosquito bites, clinical symptoms, laboratory results, complications and pregnancy were collected through the cases’ physicians using a standardised form. No information was asked about sexual history of the patients in the period before onset of symptoms.

## Results

An overview of clinical and epidemiological data from the 18 cases is presented in Table 1. The mean patient age was 49 years (range 8–61, median 54 years), 12 cases were female. Of the 15 patients with reported country of birth, nine were born in the Netherlands and six in Surinam. One of the cases was reported to be pregnant; this pregnancy ended in an intrauterine foetal death. All cases were symptomatic with rash being most frequently observed (17 cases), followed by fever (16 cases) and arthralgia (13 cases). Rash was either confined to certain body parts or spread over the entire body, in 11 cases the rash was accompanied with itch. One case developed GBS, which may have been the result of ZIKV infection. Severe and persistent thrombocytopenia with bleeding complications, possibly immune mediated, was observed in one female patient, which is described in detail elsewhere [12]. All 18 subjects resided in the European part of The Netherlands. In total, 17 had travelled to Surinam during the period of most likely acquisition of the ZIKV infection and one travelled to the Dominican Republic. Travel destinations in Surinam were predominantly the northern districts, and included cities (mainly Paramaribo) as well as rural areas, including some resorts and nature reservations. The purpose of travel was visiting friends and relatives (VFR) for nine cases, holiday for eight cases, and work related for one case. Six cases had used DEET-based mosquito repellent. Six cases had obtained travel advice regarding personal protection against mosquito bites.

**Table 1:** Description of imported ZIKV infection cases in the Netherlands, November 2015–February 2016 (*n* = 18)

Sex/age (years)	Country of birth	Date of symptom onset	Date of laboratory confirmation	Laboratory results CHIKV and DENV	Country of acquisition	Travel purpose	Symptoms	Complications	Blood abnormalities	Time to resolution (days)
M/61	SU	9 Dec 2015	11 Dec 2015	Not tested	SU	VFR/holiday	Itching rash, muscle ache, arthralgia	No	Leucopenia, atypical lymphocytes	2
F/54	SU	23 Dec 2015	30 Dec 2015	CHIKV neg DENV not tested	SU	VFR	Fever, itching rash, headache, oedema	No	Elevated liver enzymes; ASAT 32 U/L, LDH 257 U/L	>14
F/31	SU	21 Jan 2016	23 Jan 2016	CHIKV neg DENV IgG pos, IgM neg, NS1 ag test neg	SU	VFR/holiday	Rash, headache, arthralgia	No	Elevated liver enzymes; γ-GT 39 U/L	7
F/60	SU	8 Jan 2016	14 Jan 2016	CHIKV neg DENV IgG pos, IgM neg, NS1 ag test neg	SU	VFR/holiday	Fever, arthralgia	Yes, GBS	Elevated liver enzymes; AF 128 U/L, ALAT 69 U/L, ASAT 89 U/L, LDH 366 U/L	Unknown
F/33	NL	9 Jan 2016	11 Jan 2016	CHIKV qRT-PCR neg, serology pending, DENV IgG borderline, IgM neg, NS1 ag test neg	SU	VFR	Fever, itching rash, conjunctivitis, headache, muscle ache, arthralgia	No	Leucopenia	10
F/46	NL	5 Dec 2015	9 Dec 2015	CHIKV IgG/IgM neg, DENV NS1 ag test neg	SU	Holiday	Itching rash, conjunctivitis, headache, arthralgia, oedema	No	Yes, lymphopenia, atypical lymphocytes, elevated liver enzymes: LDH 297 U/L	28
M/47	NL	8 Dec 2015	17 Dec 2015	CHIKV IgG/IgM neg, DENV IgG pos, IgM weak pos, NS1 ag test neg	SU	Work	Fever, itching rash, conjunctivitis, arthralgia, muscle ache	No	Yes, atypical lymphocytes	21
F/53	NL	27 Dec 2015	30 Dec 2015	CHIKV n.a., DENV NS1 ag test neg	SU	Holiday	Fever, rash, conjunctivitis, headache, muscle ache, oedema	No	Yes, leucopenia, lymphopenia	14
M/8	NL	3 Feb 2016	4 Feb 2016	CHIKV n.a., DENV IgG/IgM neg, NS1 ag test neg	DO	VFR/holiday	Fever, rash, headache	No	Yes, leucopenia	10
F/61	NL	27 Nov 2015	1 Dec 2015	CHIKV IgG/IgM neg, DENV IgG neg, IgM weak pos, NS1 ag test neg	SU	Holiday	Itching rash, conjunctivitis, arthralgia	No	Yes, atypical lymphocytes	28
F/54	SU	17 Jan 2016	19 Jan 2016	CHIKV IgG/IgM neg DENV IgG neg, IgM weak pos, NS1 ag test neg	SU	Holiday	Fever, itching rash, conjunctivitis, arthralgia, oedema	Yes, thrombocytopenia	Yes, leucopenia, lymphopenia, atypical lymphocytes	28
F/56	SU	24 Dec 2015	30 Dec 2015	CHIKV n.a., DENV n.a.	SU	Holiday	Fever, rash, arthralgia, muscle ache, oedema	No	Yes, atypical lymphocytes	56
M/59	Unknown	24 Dec 2015	30 Dec 2015	CHIKV n.a., DENV n.a.	SU	Holiday	Fever, itching rash, conjunctivitis	No	Atypical lymphocytes	7
F/61	Unknown	4 Feb 2016	10 Feb 2016	CHIKV IgG/IgM neg, DENV IgG/IgM neg, NS1 ag test neg	SU	Holiday	Fever, rash, conjunctivitis, arthralgia	No	Lymphopenia	Unknown
F/60	NL	28 Nov 2015	2 Dec 2015	CHIKV IgG/IgM neg, DENV IgG/IgM neg, NS1 ag test neg	SU	Holiday	Fever, itching rash, arthralgia, muscle ache, oedema	No	Elevated liver enzymes: LDH 297 U/L	14
M/54	Unknown	2 Dec 2015	10 Dec 2015	CHIKV IgG/IgM neg, DENV IgG/IgM neg, NS1 ag test neg	SU	VFR	Fever, rash, arthralgia, muscle ache	No	Leucopenia, lymphopenia, atypical lymphocytes	14
M/40	NL	1 March 2016	3 March 2016	Not tested	SU	VFR/holiday	Fever, itching rash, muscle ache	No	Not measured	7
F/40	NL	1 March 2016	3 March 2016	CHIKV IgG/IgM neg, DENV IgG/IgM neg, NS1 ag test neg	SU	VFR/holiday	Fever, itching rash, muscle ache, arthralgia, oedema	No	Leucopenia, atypical lymphocytes	Unknown

*M* male, *F* female, *CHIKV* Chikungunya virus, *DENV* Dengue virus, *SU* Surinam, *NL* the Netherlands, *DO* Dominican Republic, *NS1 ag test* nonstructural protein 1 antigen test, *neg* negative, *pos* positive, *qRT-PCR* quantitative real-time polymerase chain reaction, *VFR* visiting friends and relatives, *GBS*, Guillain–Barré syndrome, *ASAT* aspartate amino transferase, *LDH* lactate dehydrogenase, *γ-GT* gamma-glutamyl transpeptidase, *AF* alkaline phosphatase, *ALAT* alanine aminotransferase, *U/L* units per litre

Onset of symptoms was abroad, on the day of return to the Netherlands and after return to the Netherlands (range 1–7 days) for four, six and eight cases, respectively. Laboratory confirmation of one case was performed in Surinam, by qRT-PCR. The period between onset of symptoms and laboratory confirmation ranged from 1 to 9 days, with a mean of 4 days. Underlying illness was reported in four patients and included a history of breast cancer, diabetes, hypertension and multiple sclerosis. Laboratory results for chikungunya virus, either qRT-PCR or serology, were available in 12 patients and were all negative. Dengue virus laboratory results were available in 13 cases (11 patients with both antibody test and nonstructural protein 1 (NS1) antigen test, two with only NS1 antigen test). Of these, four patients were dengue IgG positive (of which one equivocal), and three were weakly IgM positive. Eight patients were known to have been vaccinated against yellow fever. Blood abnormalities were found in all cases; (discrete) leucopenia as well as elevated liver enzymes was observed in five cases. Time to resolution was known for most patients, ranging from 2 days to 8 weeks, and it depended on severity of symptoms.

## Discussion and conclusion

We report 18 cases of ZIKV infections imported into the Netherlands. Most cases had a self-limiting course of disease, with the exception of a patient with GBS and a patient with thrombocytopenia and subcutaneous bleedings. Underlying illness was reported for the GBS case. The NS1 antigen test, which is a useful tool for detection of acute dengue infections, was negative in all 13 cases tested for dengue. Among the 13 cases tested for dengue, only three had a weak-positive dengue IgM result. Cross-reactivity of antibodies against other flaviviruses mainly with dengue virus infection has been well documented [13]. Several patients had elevated liver enzymes suggesting involvement of the liver by the ZIKV infection, which is also frequently seen in dengue virus infections [14]. Information about the sexual history of the patients in the period before onset of symptoms was not ascertained in this study. Seventeen cases were imported from Surinam, consistent with the high rate of transmission reported there [3]. Due to historic ties between the countries, travel to Surinam from the Netherlands is relatively frequent and the duration of stay is comparatively long. These ties also exist for the Dutch Caribbean; however, during the Christmas holidays when travel was frequent, there was no evidence of ZIKV in these areas yet. Kramer et al. (2008) found that 60 % (1159/1938) of migrants from Surinam and the Dutch overseas territories, participating in their study and residing in the Netherlands, had travelled to their country of origin during the preceding 5 years [15]. The majority of patients described (11 of 16 with information) was visiting friends and relatives (VFR) and/or were born in Surinam. International studies suggest that VFR travellers experience a higher risk of contracting travel-related infectious diseases compared to other groups of international travellers. VFR travellers are less likely to obtain pre-travel medical advice, usually have closer contact with local populations and their associated housing conditions, and are more likely to have a longer duration of travel [16, 17]. Several other European countries have reported importation of ZIKV infection cases [8, 9]. A number of cases had used DEET-based mosquito repellents. Whether infection in these cases was caused by improper use of the repellent or ineffectiveness of the repellent itself, could not be determined. Local transmission of ZIKV is considered unlikely in the Netherlands due to suboptimal climate conditions and lack of establishment of the major ZIKV vector *Ae. aegypti* and the potential vector *Ae. albopictus* [18]. However, incidental transmission of ZIKV by sexual transmission could be possible. Given the presence of *Ae. aegypti* and *Ae. albopictus* in other parts of Europe and the past outbreaks of chikungunya and dengue in various European countries, small outbreaks of ZIKV infections cannot be excluded in these areas in the future [19].

The emergence of ZIKV in South and Central America, the possible association with birth defects and neurological conditions, and the PHEIC statement of the WHO requires a comprehensive and fast public health response. Globally, the key research priority is to establish whether ZIKV has a causal relation with the occurrence of birth defects and neurological complications. Additionally, the possible sexual transmission of ZIKV as described in various case reports requires further research and diagnostic evidence to determine the probability of this transmission route [20]. In the Netherlands, multiple interventions in terms of preparedness, prevention and control of ZIKV infections are being implemented. These involve development of guidelines for testing of pregnant women with exposure to ZIKV in endemic areas during gestation, and guidelines for follow-up and clinical management of cases with abnormalities during pregnancy. Mosquito control activities in the Dutch overseas territories are ongoing. From a microbiologic perspective, diagnostics and testing algorithms need to be improved by determining the kinetics of ZIKV in clinical samples, and the development of a ZIKV-specific serological assay to identify current and past infections and distinguish these from other flavivirus infections. Other challenges include the optimal surveillance of ZIKV infections and their possible complications as well as providing information about the current global ZIKV situation and the need for pre-travel advice to the general public especially pregnant women and women intending to become pregnant (www.rivm.nl). Increasing pre-travel awareness of infectious diseases among travellers, especially VFR travellers, might enhance use of personal protection against mosquito bites (clothing, bed nets, and mosquito repellents).

## Appendix

Dutch ZIKV study team: Academic Medical Center, University of Amsterdam: A Goorhuis, MP Grobusch, J Schinkel; Erasmus Medical Center, Rotterdam: AA van der Eijk, MP Koopmans, SD Pas, CB Reusken; Harbour Hospital, Rotterdam: PJJ van Genderen; M de Mendonça Melo; Leiden University Medical Centre, Leiden: LG Visser; National Institute for Public Health and the Environment, Bilthoven: MAH Braks, JT van Dissel, JW Duijster, SJM Hahné, JHTC van den Kerkhof, JL Murk (also: University Medical Center, Utrecht), JH Reimerink, B Rockx, MA van der Sande, I Schreuder, CM Swaan, A Timen, MJ te Wierik.
